# One-Pot Synthesis of Semiconducting Quantum Dots–Organic Linker–Carbon Nanotubes for Potential Applications in Bulk Heterojunction Solar Cells

**DOI:** 10.3390/molecules28237702

**Published:** 2023-11-22

**Authors:** Mallika Dasari, Baleeswaraiah Muchharla, Saikat Talapatra, Punit Kohli

**Affiliations:** 1 School of Chemical and Biomolecular Sciences, Southern Illinois University, Carbondale, IL 62901, USA; mallikachem14@gmail.com; 2 School of Physics and Applied Physics, Southern Illinois University, Carbondale, IL 62901, USA; balu167@siu.edu

**Keywords:** semi-conducting quantum dots, interfacial molecular engineering, multi-walls carbon nanotubes, solar cells

## Abstract

Materials and composites with the ability to convert light into electricity are essential for a variety of applications, including solar cells. The development of materials and processes needed to boost the conversion efficiency of solar cell materials will play a key role in providing pathways for dependable light to electric energy conversion. Here, we show a simple, single-step technique to synthesize photoactive nanocomposites by coupling carbon nanotubes with semiconducting quantum dots using a molecular linker. We also discuss and demonstrate the potential application of nanocomposite for the fabrication of bulk heterojunction solar cells. Cadmium selenide (CdSe) quantum dots (QDs) were attached to multiwall carbon nanotubes (MWCNTs) using perylene-3, 4, 9, 10-tetracarboxylic-3, 4, 9, 10-dianhydride (PTCDA) as a molecular linker through a one-step synthetic route. Our investigations revealed that PTCDA tremendously boosts the density of QDs on MWCNT surfaces and leads to several interesting optical and electrical properties. Furthermore, the QD–PTCDA–MWCNTs nanocomposites displayed a semiconducting behavior, in sharp contrast to the metallic behavior of the MWCNTs. These studies indicate that, PTCDA interfaced between QDs and MWCNTs, acted as a molecular bridge which may facilitate the charge transfer between QDs and MWCNTs. We believe that the investigations presented here are important to discover simple synthetic routes for obtaining photoactive nanocomposites with several potential applications in the field of opto-electronics as well as energy conversion devices.

## 1. Introduction

Quantum dot-based photovoltaic (PV) materials/devices explored recently have tunable band gaps and emission properties [1,2]. Their photostability and extinction coefficient are significantly larger than that of organic dyes [3,4,5,6]. The solution processability of large-scale high-quality QDs makes them useful components for many applications, for example, in BHJSCs [7,8]. BHJSCs are promising candidates for the generation of energy utilizing solar radiation [9,10,11,12]. BHJSCs are popular due to their large electron donor (D) and electron acceptor (A) interface area, which is formed by simple blending of donor and acceptor [13]. For example, a QD-based PbS/TiO_2_ heterojunction solar cell architecture exhibited a PCE of 5.1% [7]. Similarly, inorganic–organic heterojunction solar cells with TiO_2_/Sb_2_S_3_/P3HT/Au cell structure exhibited ~5.13% PCE [14]. The dual nature of QDs as a light harvesting material and electron transporting material is due to the easily tunable opto-electronic properties [15,16]. Blends of CdSe/MEH-PPV and CdSe/P3HT nanocomposite-based BHJSC exhibited a PCE of 1.7% and 2.6%, respectively [17,18].

BHJSCs were first reported in 1995 by A. J. Heeger’s group, where the authors demonstrated that blended poly(2-methoxy-5-(2′-ethyl-hexyloxy)-1,4-phenylene vinylene) (MEH-PPV) and fullerenes (C_60_) exhibited a carrier collection efficiency and a PCE of 29% and 2.9%, respectively [19]. Over the past years, extensive research has led to the improved performance of BHJSCs [20]. The nanoscale crystalline domains with improved hole mobility and light absorption in the active layer make excellent materials for BHJSC application [21]. The BHJSC fabricated using poly(3-hexylthiophene) (P3HT) and [6,6]-phenyl-C61-butyric acid methyl ester (PC_61_BM) system showed a PCE of more than 5% [22].

The excitons generated far from the donor–acceptor interface, with limited exciton diffusion length (a few nanometers for conjugated polymers, <20 nm) [2] and exciton lifetime (<50 ns) [23], may result in exciton recombination. The electron–hole combination yields photoemission and results in the decrease in PCE. When the D–A interface is within the exciton diffusion length, the excitons may find one or multiple D–A interfaces in their close vicinity that may reduce the recombination losses [2,24]. Charge transport through continuous conductive pathways in D–A bulk matrix to electrodes aid in improving the PCE.

**Figure 1:**
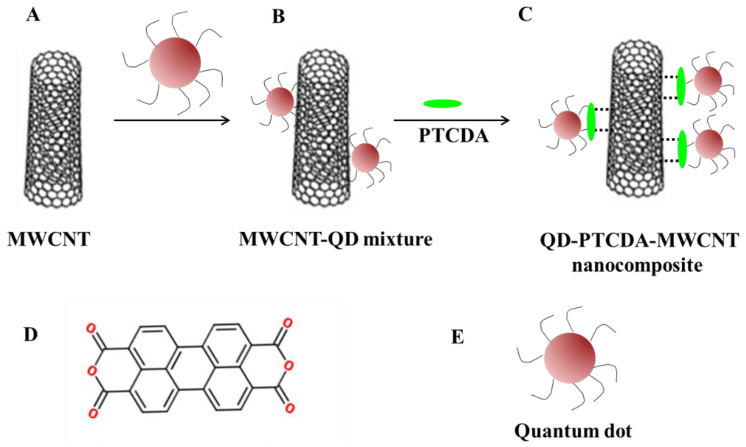
Schematic of QD–PTCDA–MWCNTs nanocomposite. MWCNTs (**A**) were added to a QD solution during QD synthesis (**B**). (**C**) QD–PTCDA–MWCNTs nanocomposite was formed through the addition of PTCDA molecules to the QD–PTCDA solution, which acted as a molecular linker between MWCNTs and QDs. “^…^”in (**C**) denote π–π interactions between PTCDA and MWCNTs, whereas the green oval represents the PTCDA molecule. (**D**) The chemical structure of a PTCDA. (**E**) A schematic representation of QDs.

**Figure 2:**
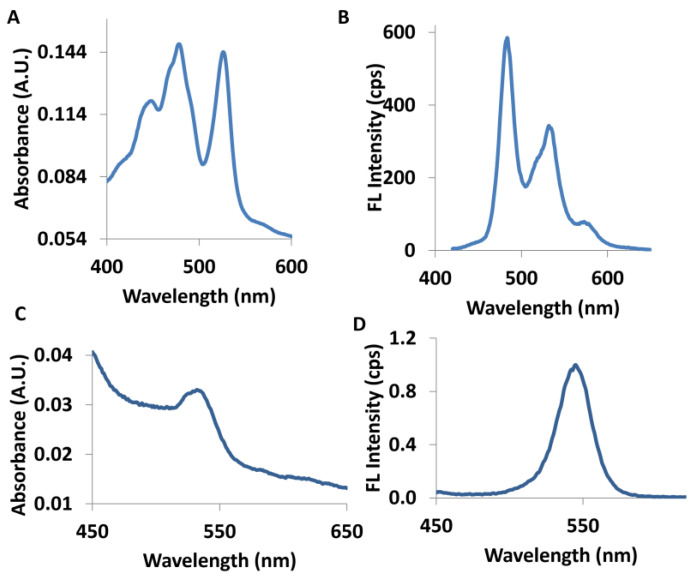
UV-Visible absorption (**A**) and fluorescence emission (**B**) spectra of the PTCDA molecule in dimethyl sulfoxide (DMSO). UV-Visible absorption (**C**) and fluorescence emission (**D**) spectra of the QDs in chloroform solution.

In this manuscript, QD–PTCDA–MWCNTs photoactive nanocomposites were synthesized in a single one-pot step, which does not require multistep sequential synthesis and purification of QD–PTCDA–MWCNTs nanocomposites. This also minimizes chemical waste. Overall, the material synthesis of the DA active material demonstrated in this report is less labor intensive; therefore, saves time and energy for the production of photoactive nanocomposites for device fabrication. The QD–PTCDA–MWCNTs nanocomposites possess two interfaces—covalent attachment of PTCDA to QDs and π–π stacking between PTCDA and MWCNTs (Figure 1). Incorporation of PTCDA, as a molecular linker, in the QD–PTCDA–MWCNTs nanocomposite significantly improved the binding interactions between QDs and MWCNTs—~100-fold increase in the QD attachment on MWCNTs in the presence of PTCDA was observed in the electron microscopy studies. PTCDA is an π-conjugated organic molecule with absorption and emission in the visible spectrum region (Figure 2). PTCDA also acted as an electron acceptor for a photoexcited QD donor in the nanocomposites [25,26]. The photoinduced charge transport of the nanocomposites was investigated using the devices fabricated with QD–PTCDA–MWCNTs nanocomposites.

## 2. Results and Discussion

The electron donor–acceptor morphology of the active materials in BHJSCs is crucial to controlling its interface [27,28], and it depends on a number of factors including, solvent evaporation rate, solubility and compatibility of donor and acceptor in the solvent used for processing, and substrate surface chemistry and morphology. An intimate D–A interaction may facilitate efficient charge dissociation and transport to charge collecting electrodes [28]. The main objective of this work is to synthesize and characterize photoactive nanocomposites in a single pot and to study the photoinduced charge transport in the nanocomposites. The electron donor and electron acceptor in the nanocomposite were intimately linked through a covalent bond. Another important question we intend to investigate is the role of the organic linker in the active material. We chose PTCDA because the reactive anhydride groups of PTCDA can readily form covalent bonds with amine functionalized QDs, providing close contact between them. Furthermore, fused phenyl rings of PTCDA and MWCNTs can interact through π–π stacking. Therefore, PTCDA bridges QDs and MWCNTs, which presumably aided in facilitating the charge transfer from photoexcited QDs to MWCNTs and collecting electrodes.

### 2.1. Infrared Spectroscopy Characterization

**Figure 3:**
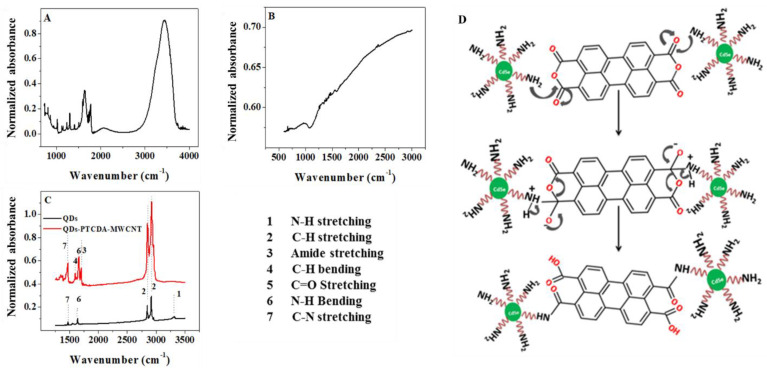
FTIR characterization of QDs, PTCDA, MWCNT, and composite. (**A**) IR of PTCDA depicting C=O stretching peak at 1770 cm^−1^ from cyclic anhydride of the PTCDA molecule. (**B**) IR spectrum of MWCNTs. (**C**) The black IR absorbance spectrum of the QDs multiple peaks corresponding to N–H bending, N–H stretching, and C–H bending. The red IR spectrum of the QD–PTCDA–MWCNTs nanocomposite, that depicted C=O stretching at 1664 cm^−1^ from the amide bond, confirmed the formation of covalent bonds between QDs and PTCDA in the nanocomposite. (**D**) The proposed reaction mechanism for covalent bonding between amine containing QDs and anhydride groups of PTCDA molecule, yielding amide linking between QDs and PTCDA.

FTIR spectroscopy is a powerful analytical technique for qualitative and quantitative structural analysis of organic and inorganic molecules. We have utilized FTIR spectroscopy for probing the QD–PTCDA interface in the nanocomposite. The nanocomposite samples for IR investigations were prepared by depositing the nanocomposites suspended in chloroform onto a KBr disc. Chloroform was evaporated and another KBr disc was placed to sandwich the samples between two KBr discs. Importantly, the peak at 1770 cm^−1^ in the FTIR spectrum of the nanocomposite originates from C=O of the anhydride rings of the PTCDA (Figure 3A, Table 1) [29]. Figure 3C shows the infrared vibrational peaks for CdSe QDs (black spectrum) and QD–PTCDA species (red spectrum). The ODA peaks at 1452 cm^−1^, 1590 cm^−1^, and 2917–2848 cm^−1^ are attributed to C–H bending, N–H bending from NH_2_, and C–H stretching from C_18_ alkyl chain, respectively [30]. ODA (C_18_-NH_2_) is believed to be interdigitated with TOPO through hydrophobic–hydrophobic interactions [31,32]. The NH_2_ groups present on the QD surface were utilized for covalent bonding with anhydride groups of PTCDA through a spontaneous reaction, yielding amide bonds at the QD–PTCDA interface. The IR spectrum in Figure 3C shows that the peaks at 1460 cm^−1^, 1592 cm^−1^, 1664 cm^−1^, and 2911–2848 cm^−1^ correspond to C–H bending, N–H bending, C=O amide stretching, and C–H stretching, respectively. Importantly, the C=O from the anhydride ring at 1770 cm^−1^ of the PTCDA disappeared, but the appearance of a new peak at 1664 cm^−1^ corresponding to amide bonds in the nanocomposites confirmed the covalent bonding between PTCDA and ODA. The proposed covalent binding of QD with PTCDA is depicted in Figure 3D.

### 2.2. Transmission Electron Microscopy (TEM) Analysis of QD–PTCDA–MWCNTs

**Figure 4:**
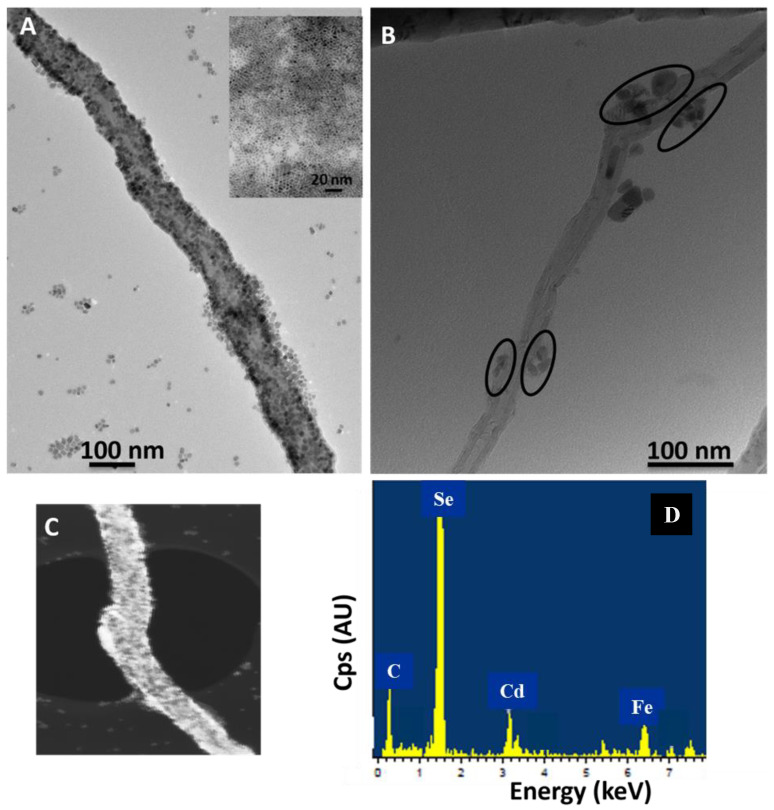
TEM images of an MWCNT functionalized with QDs in the presence (**A**) and absence (**B**) of the PTCDA. The QD diameter = 5.2 nm ± 1.4 nm (n = 22). (**C**) A backscattering electron (BSE) image of the nanocomposite where the brighter areas indicate the presence of the QDs. (**D**) An EDS spectrum of the nanocomposites showing elemental X-ray analysis of C K*_a_* at 0.277 keV, Fe K*_a_* at 6.398 keV, Cd L*_a_* at 3.133 keV, and Se L*_a_* at 1.379 keV.

Figure S2 shows a TEM of the CdSe QDs prepared by the solution-phase method but without the addition of MWCNTs and PTCDA in the reaction vessel. Figure 4A show a TEM image of QDs attached on MWCNTs in the presence of PTCDA. The TEM images indicate that the QDs are well distributed on MWCNTs, and that they were adhered strongly to the MWCNTs. On the other hand, Figure 4B shows a TEM of the nanocomposites prepared in the absence of PTCDA. The highlighted areas in the circles are QDs present on the MWCNTs surface in the absence of PTCDA. The significant decrease in QDs density on MWCNTs in the absence of PTCDA confirmed the importance of PTCDA as a linking molecule between the QDs and MWCNTs.

We estimate the QD attachment density on the MWCNT surface with and without the presence of PTCDA linker molecule. The area of MWCNT in Figure 4A,B was estimated to be 4.9 × 10^−13^ m^2^ and 7.2 × 10^−14^ m^2^, respectively. The average number of QDs functionalized in the presence of PTCDA on a 1 μm × 0.1 μm (length × diameter of the MWCNTs) was ~2600, whereas only ~20–25 QDs were observed in the absence of QD functionalization with PTCDA. Specifically, ~100-fold increase in the QD attachment on MWCNTs was observed following the covalent functionalization of QDs with PTCDA. These results are consistent with our previous report, where in the presence of a linker organic molecule ethanethiol-perylene tetracarboxylic diimide (ETPTCDI, which is similar in chemical structure to PTCDA), a large enhancement in the QD attachment to MWCNTs was exhibited, whereas the observed QD density on MWCNTs in the absence of ETPTCDI was significantly lower [36]. Backscattering electron imaging (BSE) measurements also exhibited a large contrast in the micrograph due to QD attachment to MWCNTs (Figure 4C), where brighter areas in the micrograph correspond to heavier atomic elements (Cd and Se). Overall, these results confirmed a significant enhancement in the attachment of the QDs on MWCNTs by utilizing molecular interfacial engineering through the employment of a molecular linker between MWCNTs and QDs.

The elemental composition measurements using EDS showed the elemental Cd, Se, and C peaks corresponding at 3.133 keV, 1.379 keV, and 0.277 keV, respectively (Figure 4D). Whereas the elemental signals of Cd, Se, P, and O, in the EDS spectrum originated from CdSe QDs, the carbon signal is expected to come from QDs, MWCNTs, and organic ligands in the nanocomposites. Overall, our TEM, BSE, and EDS measurements indicated that the QDs density on the MWCNTs in the presence of PTCDA was significantly increased as compared to nanocomposites in the absence of the molecular linker PTCDA. These results conclusively confirmed the significance of an appropriate molecular linker at the QD–MWCNTs interface, which significantly enhanced the concentration of QDs on MWCNTs through short-order molecular interactions.

### 2.3. Thermogravimetric Analysis (TGA)

**Figure 5:**
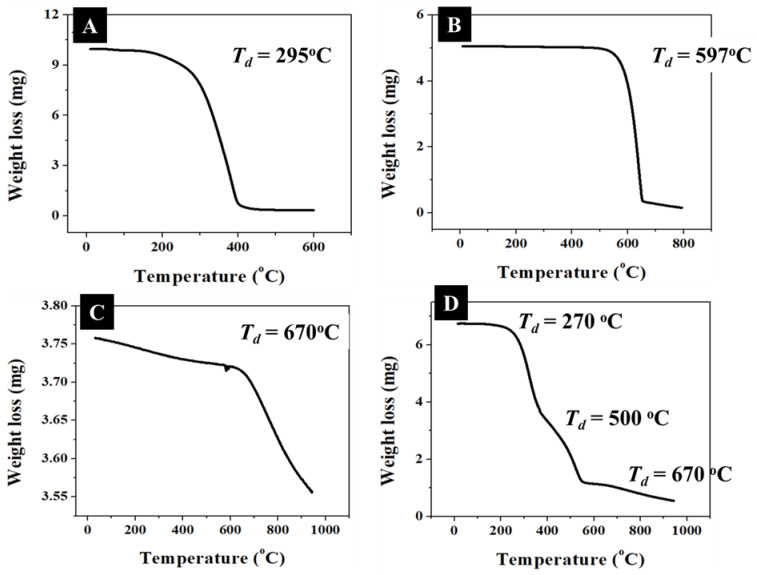
Thermogravimetric analysis of PTCDA (**A**), QDs (**B**), and MWCNTs (**C**) showed major weight loss at the decomposition temperature (*T*_d_) ~295 °C, 597 °C, and 670 °C, respectively. (**D**) TGA analysis of the nanocomposite showed three decomposition steps with *T*_d_ = 270 °C, 550 °C, and 670 °C.

About 6.7 mg of nanocomposite was heated from 20 °C to 950 °C at a rate of 20 °C/min under the flow of N_2_ gas. TGA analysis of the individual components was also performed to determine the decomposition rate and weight loss. TGA analysis provided quantitative decomposition information of individual components. In these experiments, the apparent decomposition temperatures (*T*_d_) for the PTCDA and QDs were ~295 °C and 597 °C, respectively (Figure 5A,B), whereas only ~5% of MWCNTs initial mass was observed to decompose at ~670 °C (Figure 5C). Importantly, the weight loss for PTCDA, QDs, and MWCNTs showed different decomposition regions of 120–400 °C (Figure 5A), 480–656 °C (Figure 5B), and 608–950 °C (Figure 5C), respectively, without any significant overlap between them, which allowed for an accurate gravimetric analysis of the nanocomposites. The TGA analysis of the nanocomposite showed three major steps with *T*_d_ of ~275 °C, 551 °C, and 656 °C, representing decomposition temperatures of low molecular weight organic species (TOPO, ODA, and PTCDA), QDs, and MWCNTs, respectively. The individual component ratio present in nanocomposite was estimated using the decomposition curve in Figure 5D. Due to the sufficient separation in the mass loss—temperature profiles for organic, QDs, and MWCNTs, the TGA data provided a convenient way of estimating the mass of various species in the nanocomposites. The weight loss ratio for the organic species:QD:MWCNTs in the nanocomposite was estimated to be 76:51:1. Whereas the weight loss for the MWCNTs was minimum (<1.5%) for 25–950 °C region, which suggested that the weight loss of the organic molecules contributed to >99% of the total weight loss in the 25–390 °C region in the nanocomposites. Therefore, for the present discussion, we neglect the contribution of weight loss due to MWCNTs to the total weight loss. Considering a complete coverage of QDs (average diameter = 5 nm) with a close packing of the organic species, we estimate organic-to-QD mass ratio of ~0.5. However, the experimental organic-to-QD mass ratio of ~1.5, which is about three times larger than that of the estimated organic-to-QD mass ratio value. This simple analysis suggests that the QD–PTCDA–MWCNTs nanocomposite likely contains additional organic molecules than that is presumed in the calculations. The absence of an accurate compositional ratio of different organic species (PTCDA, ODA, and TOPO) due to their similar thermogravimetric response, adds to the uncertainty in the estimation of accurate weight loss ratio of organic molecules to QDs. Furthermore, the estimation of the expected organic-to-QD mass ratio involved close packing of molecules on a flat surface of the same surface area as that of 5 nm diameter QDs surface area, which underestimates the organic molecule to QDs mass loss. Finally, the ratio of three major organic molecules was assumed to be 1:1:1, which is likely not the case in the experimental samples. Therefore, although the TGA measurements provided useful compositional information; we do not overemphasize mass loss differences between the experimental TGA results and that of the expected results based on geometric considerations. These results should be treated with caution.

### 2.4. Photophysical Quenching Studies of QD–PTCDA–MWCNTs Nanocomposites

**Figure 6:**
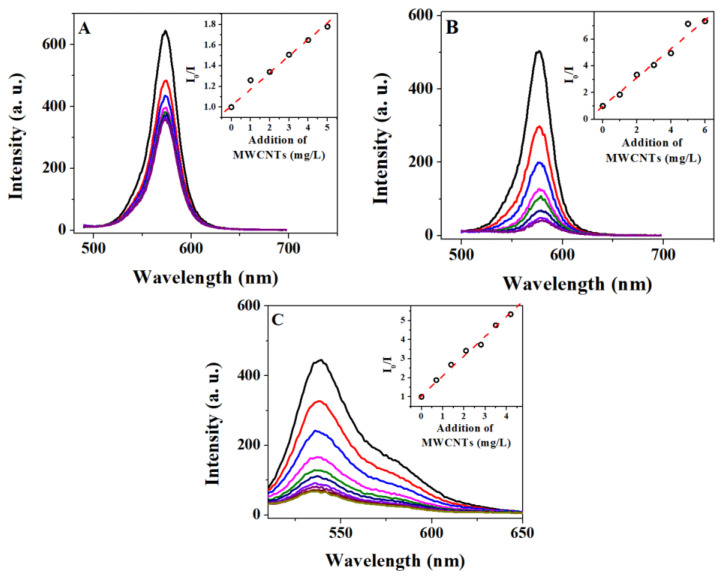
Fluorescence quenching and Stern–Volmer measurements. The emission quenching of QDs for the QDs/MWCNTs (**A**) and the QDs–PTCDA/MWCNTs (**B**). (**C**) The emission quenching of PTCDA for the PTCDA–MWCNTs pair. The concentrations of QDs, PTCDA, and MWCNTs used for quenching experiments were 50 nM, 50 nM, and 0.01% *w*/*w*, respectively. The *K_sv_* value estimated from the *I*/*I*_0_–[MWCNTs] plot (insets) of QDs/MWCNTs (**A**) was ~7 times smaller than that of the QDs–PTCDA/MWCNTs nanocomposites (**B**). The increase in *K_sv_* indicated the presence of PTCDA enhanced fluorescence emission quenching through intimate QD–MWCNTs contact. Excitation wavelengths for QDs and PTCDA were 400 nm and 490 nm, respectively.

Photophysical fluorescence quenching experiments for the QD–PTCDA–MWCNTs nanocomposites were performed to study the effect of PTCDA on photophysical properties of QDs and that of nanocomposites. We anticipate quenching of the QD emission is expected to be higher when QDs are intimately attached to the MWCNTs through PTCDA, as a molecular linker. This is due to enhanced electron and/or energy transfer between QDs and MWCNTs or through formation of ground-state dark donor-acceptor complexes. Stern–Volmer relationship was used to estimate the fluorescence emission quenching efficiency between donors and acceptors using $\frac{I_{0}}{I}$ = 1 + *K_sv_* [*Q*]. Here, *I*_0_ and *I* represent the donor emission intensity in the absence and presence of quencher, respectively [37], and *K_sv_* is the Stern–Volmer constant which was obtained from the slope of the Stern–Volmer plot. *K_sv_* is a measure of quenching efficiency and provides the accessibility of acceptor to donors—higher *K_sv_* represents more efficient donor emission quenching by the acceptors. *K_sv_* values for four different D/A pairs are given in Table 2. Fluorescence quenching constant (*K_sv_*) for the QDs and MWCNTs pair was ~7 times larger in the presence of PTCDA than in the absence of PTCDA (Table 2 and Figure 6). QD/MWCNTs, QD/PTCDA, PTCDA/MWCNTs, and QD–PTCDA/MWCNTs pairs exhibited *K_sv_* values of 0.16, 0.25, 1.05, and 1.09, respectively (Table 2). Whereas the *K_sv_* for QD/MWCNTs pair was lowest of the four pairs studied, the highest *K_sv_* observed values were for PTCDA/MWCNTs and QD–PTCDA/MWCNTs pairs, which were >6 times larger than in the absence of MWCNTs. These results implied significantly enhanced quenching of the donor species (PTCDA and QD–PTCDA pair) to MWCNTs (acceptor), possibly either through the formation of ground state complexes (QD–MWCNTs and QD–PTCDA–MWCNTs) or through quenching of the excited-state donors to the MWCNTs. The fact that the presence of PTCDA for both the QD–PCTDA/MWCNTs and PTCDA/MWCNTs pairs showed the highest *K_sv_* values confirmed strong molecular interactions between QDs and MWCNTs in nanocomposites mediated through PTCDA molecular linkers. These results also implied the significance of PTCDA as a molecular linker for energy- and charge-transfer processes for nanocomposites created with QDs and MWCNTs [38,39]. Overall, the quenching studies agree well with the results and conclusions obtained from IR, TEM, and EDS data.

### 2.5. Electrical Transport Characterization of the QD–PTCDA–MWCNTs Nanocomposites

**Figure 7:**
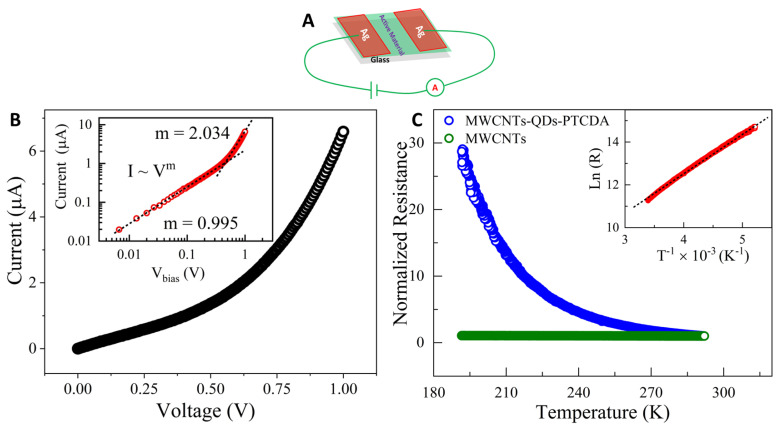
(**A**) Schematic of the photodetector device fabricated with QD–PTCDA–MWCNTs nanocomposites. (**B**) Nonlinear current–voltage behavior was observed for the prepared device with an applied bias. Inset shows the log–log plot of current–voltage curve, in which at lower bias, the sample displays ohmic behavior and it changes to space charge limited currents at higher bias. (**C**) Normalized electrical resistance versus temperature of MWCNTs and QD–PTCDA-MWCNTs nanocomposite plotted in the range of 190–300 K. Inset shows the plot of natural logarithm of resistance ln(R) versus inverse of temperature (*T*^−1^) of the QD–PTCDA-MWCNTs nanocomposite plotted in the range of 190–300 K.

Room temperature current–voltage (I–V) characteristics were carried out on a two-terminal device fabricated using QD–PTCDA–MWCNTs nanocomposites (Figure 7A). Figure 7B displays the current response observed with varied applied electric potential bias. At a bias below 0.5 V, the device displayed ohmic behavior where the current is directly proportional to the applied bias (inset of Figure 7B). As the bias was increased, the current response deviates from the ohmic nature and follows a power law [40], of the form *I* α *V*^2^ (inset of Figure 7B). Typically, this type of behavior is seen in space charge limited currents (SCLC), where the current is governed by the Mott–Gurney equation [41]: *J* $=\frac{9}{8}\mu \epsilon_{0}\epsilon_{r}\frac{V^{2}}{L^{3}}$. Here, *J* is the current density, *μ* is the charge-carrier mobility, $\epsilon_{0}$ is the free-space permittivity, $\epsilon_{r}$ is the dielectric constant, *V* is the applied voltage, and *L* is the separation between the contacts.

Temperature dependence of the electrical resistance over a temperature range (190 K ≤ T ≤ 300 K) was measured for MWCNTs and QD–PTCDA–MWCNTs samples (Figure 7C). The temperature dependence of resistance curve for the MWCNTs displayed a metal-like behavior, whereas the QD–PTCDA–MWCNTs sample showed a semiconducting behavior. These behaviors are consistent with material properties of MWCNTs and that of QD–PTCDA–MWCNTs nanocomposites. Importantly, I–V curves for the QD–PTCDA–MWCNTs nanocomposites suggested that MWCNTs were covered with semiconducting material on their surface and that there was little or minimum ohmic MWCNTs–MWCNTs electronic pathways in the devices. The electrical resistance measured for the QD–PTCDA–MWCNTs sample increased slowly with the decreasing temperature. The resistance of the device was observed to be 80.4 kΩ at 300 K, which was increased to 2.46 MΩ at 190 K. For further analysis, the data were fitted using the Arrhenius model for the temperature dependence of resistance, which is governed by the equation: *R*(*T*) = *R*_0_ exp(*E_g_*/*k_B_T*), where *E_g_* is the activation energy of the charge transport in the devices, and *k_B_* is the Boltzmann constant [42]. Natural logarithm of resistance ln(*R*) versus inverse of temperature (*T*^−1^) was plotted as shown in the inset of Figure 7C. ln(*R*) versus *T*^−1^ plot was best fit to a straight-line emphasizing bandgap dominated Arrhenius-like temperature dependence. The linear fit of the data shown in Figure 7C inset yielded an activation energy of the order of 165 meV. The activation value obtained in the experiments is of a similar order of magnitude to those for 2D materials. For example, the activation energy on the order of 70 meV was obtained for the reduced graphene oxide. The temperature dependent electrical transport of disordered reduced graphene oxide [42] and individual B-doped nanotubes exhibited an activation energy of 55–70 meV [43]. Similarly, Johnston et al. have shown that the activation energy values for carbon nanotubes can be ~150 meV [44].

### 2.6. Photoinduced Current Characterization

**Figure 8:**
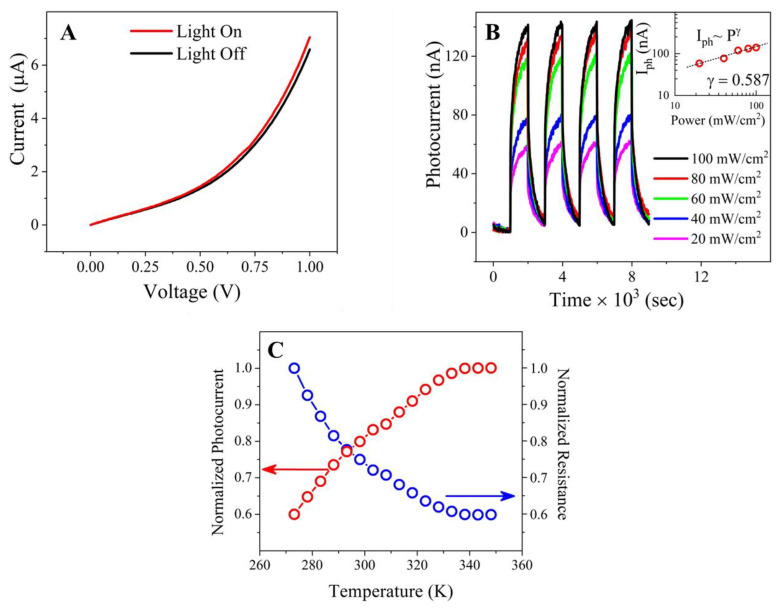
(**A**) Current–voltage characteristics of QD–PTCDA–MWCNTs device with and without illumination of light (device area ~5 cm^2^, *P* = 130 mW/m^2^). (**B**) Photocurrent–time dependence of the QD–PTCDA–MWCNTs nanocomposites plotted at various power values of the incident light. The light intensity was varied by changing the distance between the light source and the device. Inset shows the plot of photocurrent versus power of the incident light which fits to a fractional power-law fitting. (**C**) The data show the increase in photocurrent in the temperature range of 273–350 K under the light intensity of 130 mW/m^2^.

The photoinduced charge generation characteristics of the QD–PTCDA–MWCNTs devices were characterized by photoexcitation of donors in a two-electrode device using a Newport solar simulator (model 67005) under AM 1.5 conditions. Figure 8A displays the observed current response with an applied bias under light ON and OFF conditions. The nonlinear behavior of the photoinduced current (*I*_ph_) was seen with an increase in voltage in both cases of light ON and OFF. *I*_ph_ for the devices created with QD–PTCDA–MWCNTs was enhanced by ~130% when the photon power intensity was increased from 20 mW/m^2^ to 100 mW/m^2^ (Figure 8B). *I*_ph_ as high as 140 nA was observed with an illumination power of 130 mW/m^2^. The dependence of the *I*_ph_ on the light power intensities *P* (20 mW/m^2^ ≤ *P* ≤ 100 mW/m^2^) showed fractional power dependence of the form of *I_ph_*~*P^γ^* with *γ* = 0.587 (inset of Figure 8B). The power exponent *γ* can shed light on the various recombination mechanisms that are associated during the photoconduction process. *I*_ph_ response in disordered semiconductors typically follows a fractional power law with power exponent *γ* between 0.5 and 1 (0.5 < *γ* <1). Furthermore, *γ* value of close to 1 denotes monomolecular recombination processes and a value of *γ* = 0.5 is associated with bimolecular recombination processes. Broadly speaking, however, any fractional value of *γ* is associated with the presence of the modulation of trap states and implicates their role in the recombination processes that occur during the charge-conduction process [45]. Typical fractional *γ* values that are reported for a variety of low dimensional materials range from 0.25 in graphene [46] to 0.4 in single layer WS_2_ [47]. These mechanisms are also demonstrated in other photoactive 2D materials, as well [47,48]. Another plausible explanation for the observed low power exponent values measured on devices in the studies is the manifestation of photogating effect, i.e., the substrate generated photovoltage that can act as an electrical gate on the measured device which may modulate trap states [49,50]. We believe that the fractional *γ* values seen in our measurements are due to the presence of trap states in the composite materials originating from defects, disorders, and dissimilar interfaces that are being created during the synthesis process.

### 2.7. Temperature-Dependent Photocurrent Characteristics

In a semiconductor, *I*_ph_ depends on both the illumination power and temperature. An increase in the temperature may enhance *I*_ph_ due to thermal agitation, which provides energy to overcome the barrier for photoconduction [51]. At high temperatures, however, the reverse can be true. Specifically, at elevated temperatures, the internal collision of charges with atoms increases, resulting in the enhanced recombination of charges [52]. The charge collisions lead to an increase in the resistance for charge propagation. The photocurrent–temperature characteristics of the QD–PTCDA–MWCNTs photodiodes were studied between 273 K and 350 K. The normalized *I*_ph_ showed an exponential increase (~50%) up to 338 K followed by plateau for a temperature above 338 K (Figure 8C). This behavior is attributed to the losses of charges due to the collisions at higher temperature. Similar studies performed on a graphene-based photodetector reported >50% increase in photocurrent in the range of 150–400 K [53].

### 2.8. Fabrication and Characterization of BHJSC That Comprise the QD–PTCDA–MWCNTs Nanocomposite

**Figure 9:**
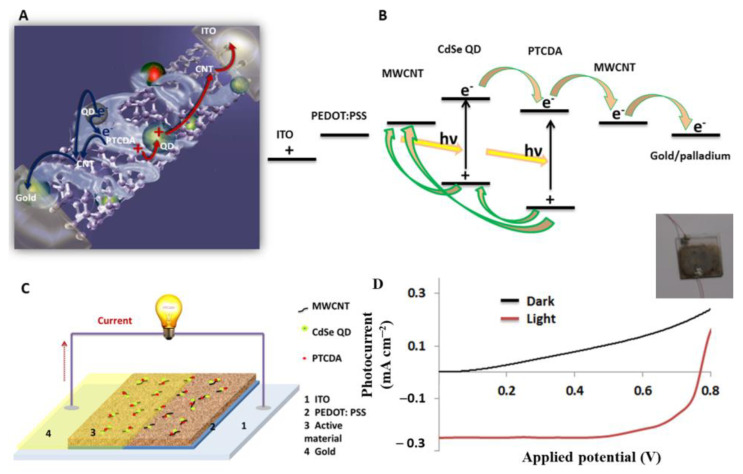
(**A**) A schematic of charge transport in QDs–PTCDA–MWCNTs nanocomposite. Although the MWCNTs in the QD–PTCDA–MWCNT nanocomposites are random, the depiction of a single MWCNTs strand in the nanocomposite in (**A**) is shown to aid the eyes. (**B**) The energy diagram showing the electron and hole transport in the nanocomposite after photoexcitation of donors. (**C**) A schematic of the BHJSC device fabricated using QDs–PTCDA–MWCNTs nanocomposite. (**D**) A typical I–V curve of the BHJSC fabricated using QDs–PTCDA–MWCNTs nanocomposites. Inset in (**D**) shows an optical photograph of a typical device. Open circuit voltage (*V_oc_*) and short circuit current (*I_sc_*) were 0.77 V and 0.25 mA cm^─2^, respectively.

Figure 9A,B show schematics of the electron transfer between individual components of the nanocomposite after photoexcitation of CdSe and PTCDA in the nanocomposite. A schematic of the BHJSC devices created with QD–PTCDA–MWCNTs nanocomposite is shown in Figure 9. In the first step, the photoexcited electrons were transferred from the excited energy level of the QDs to the bridging molecule PTCDA. The electron in the excited state energy level (LUMO) of the PTCDA was transferred to the MWCNTs. The last step in the electron transport in the nanocomposite is the extraction of electrons at the cathode (gold/palladium). Electrons from the excited level of the QDs can also be transferred directly to the MWCNTs (Figure 9B). A similar photoinduced electron generation in PTCDA can also occur where the excited state electron in the PTCDA will be transferred to MWCNTs. The holes generated in QDs were transferred to MWCNTs through the PTCDA mediator. Similarly, the holes formed in PTCDA can also be directly transferred to MWCNTs as shown in Figure 9B. Thus, the MWCNTs in the nanocomposite can carry both electrons and holes generated in the nanocomposites after photoexcitation. This is likely to contribute to a low photon–electron conversion efficiency in these nanocomposites. A thin layer of PEDOT:PSS electron blocking material was used at the anode surface to reduce electron transport to the anode surface.

A typical I–V curve of a BHJSC device fabricated using QD–PTCDA–MWCNTs nanocomposite under light illumination (AM 1.5) is shown in Figure 9D. The typical values of the short-circuit current (*I_sc_*), open-circuit voltage (*V_oc_*), fill factor (FF), and photon conversion efficiency (PCE) were 0.25 mA cm^─2^, 0.77 V, 0.43, and 0.3%, respectively. These devices exhibited FF compared to the reported FF values range of 0.4–0.7 for some organic solar cells [54]. A PV cell system comprising graphene/CdSe and graphitic carbon/CdTe showed a PCE of 1.25 and 1.36%, respectively [55,56]. The BHJSCs fabricated using a nanocomposite without PTCDA, however, displayed a much lower PCE (0.1%), suggesting that the PTCDA molecular linker plays an important role in improving the charge generation and transfer between QDs and MWCNTs. Importantly, PTCDA increased the QD concentration on MWCNTs by about two orders of magnitude as observed in the TEM measurements. Therefore, both the intimate proximity of QDs and MWCNTs through PTCDA as a bridge molecule and energetic considerations were likely to contribute to the enhanced charge transfer and charge transport between QDs and MWCNTs. These results are consistent with our previous studies where a perylenediimide derivative was used as a bridging molecule for QD and MWCNTs [36]. The typical PCE value of the QD–PTCDA–MWCNTs nanocomposite devices was ~0.3%, suggesting that only a small fraction of incident photons on the device converted into a useful photoinduced current. However, a major portion of photons were converted into heat and/or other non-electrical form. We attribute the low PCE for the devices reported here is dominated by charge recombination mechanism. The randomly dispersed MWCNTs may carry both the electrons and holes, leading to the enhanced charge recombination. Rationally designing charge transporting pathways, that would minimize the charge recombination by routing electrons and holes through separate non-communicating pathways, is likely to enhance the PCE. Since the PCE of a device also depends on the density of photoactive components and processing parameters, as well; further optimization of the density of charge generating species (for example, QDs and PTCDA) may potentially also improve the PCE. BHJSCs in the present studies were fabricated under ambient conditions, and they were not hermetically sealed to external environmental factors. The presence of oxidative agents and other reactive species can adversely affect the device performance [36]. Fabricating and processing devices in an inert atmosphere and protecting them from external species is also crucial to the long-term stability of the devices. Some of these experiments are underway and will be reported in the future.

## 3. Experimental Section

### 3.1. Materials

The 3,4,9,10-perylenetetracarboxylic dianhydride (PTCDA) was purchased from Sigma Aldrich. trioctylphosphine oxide (TOPO), octadecylamine (ODA), stearic acid (SA), cadmium oxide (CdO), selenium, trioctylphosphine (TOP), and poly(3,4-ethylenedioxythiophene) poly(styrene-sulfonate) (PEDOT:PSS) were purchased from Fisher Scientific. Ferrocene and xylene, used in the CNT growth, were purchased from Acros Organics.

### 3.2. Methods

The characterization of QDs was performed using UV-Visible spectroscopy (Perkin-Elmer Lambda 25, Shelton, CT, USA), fluorescence spectroscopy (Perkin-Elmer LS-55, Shelton, CT, USA), scanning electron microscopy (Thermo Fisher Quanta 450, Hillsboro, OR, USA), and transmission electron microscopy (Hitachi 7650, Tokyo, Japan). Temperature-dependent electrical transport data were acquired using a home-built data acquisition system comprising Janis SHI–4–1 high vacuum Closed Cycle Refrigerator System (Lake Shore, Westerville, OH, USA) and Keithley 2400 source meter (Keithley, Solon, OH, USA), both of them were controlled by LabView programming codes. The device characterization was carried out using an AM 1.5 solar simulator (Newport Inc., Deere Ave Irvine, CA, USA) with a 150 W Xenon arc lamp. The measured photon flux for Xenon lamp was 130 mW/m^2^ at 0.6 m from the source light as measured using a photodetector (Melles Griot, Carlsbad, CA, USA). Thermogravimetric analysis (TGA) was performed using a TA instrument Q50. For TGA analysis, the sample was heated to 900 °C at a heating rate of 20 °C/min under the flow of N_2_. The backscattering electron (BSE) measurements of the nanocomposites were performed using energy dispersive X-ray spectroscopy (Oxford X-Max 50, Abingdon, UK) and scanning electron microscopy (Thermo Fisher Quanta 450) by coating QD–PTCDA–MWCNTs nanocomposites on a clean glass substrate, which was thoroughly dried in the oven at 80 °C for 20 min. The dried nanocomposite film was coated with a thin conductive metal layer (~5–10 nm) using Denton Vacuum Desk III sputter coater. The conductive coating for SEM samples prevents charging of the specimen and improves the signal-to-noise ratio in the SEM imaging. For TEM analysis, the nanocomposites were deposited on a TEM grid, and the solvent was evaporated at 37 °C for ~2 h prior to imaging.

### 3.3. MWCNT Growth Procedure

The growth of the MWCNTs on SiO_2_ substrate was achieved by the air-assisted chemical vapor deposition technique [57,58,59]. A horizontal tube furnace was purged with argon gas and heated to 790 °C in an argon environment and a solution of ferrocene (catalyst precursor) and xylene (carbon source) was injected continuously into the furnace with a syringe pump at a constant rate of 12 mL/hr. The injected solution vaporized as it entered the furnace, and the vapor was carried into the reaction zone using a gas mixture of argon and hydrogen (85%/15% ratio) with a flow rate of ~400 sccm. During the growth process, a small amount of air (~2.5 sccm) was mixed with the reaction environment to maintain the catalyst activity. The reaction time was controlled by adjusting the feeding time of xylene/ferrocene solution. The quartz tube reactor was cooled down to room temperature after the growth time in the argon environment. An array of densely packed aligned MWCNTs was obtained on the SiO_2_ substrate in both horizontal and vertical directions.

### 3.4. Synthesis of Photoactive Nanocomposites

CdSe QDs were prepared using a standard synthetic procedure [60]. Octadecylamine (2.05 g) was heated in a two-neck 100 mL round-bottom flask to 150 °C for roughly 30 min to remove all the water from ODA. The flask was cooled down to room temperature, and a mixture of CdO (13 mg), SA (0.10 g), and TOPO (2.05 g) was added to the reaction flask. The flask was sealed and placed under a continuous flow of argon. The temperature of the reaction flask was raised to 300 °C. After the reaction mixture turned pale yellow, the temperature of the reaction mixture was reduced to 70 °C and MWCNTs (0.5 mg) suspended in dichloromethane (DCM) were added to it. The temperature of the QD–MWCNTs mixture was again increased to 300 °C, and Se powder (79 mg) dissolved in trioctylphosphine (1 mL) was swiftly injected into it. Following this, 1.0 g of powdered PTCDA was added to the reaction mixture and the reaction was left for 48 h. After 48 h, the nanocomposite sample was extracted with a glass pipette and air cooled to room temperature, which resulted in the product mixture to turn into a solid gel. Approximately 1 mL of chloroform was added to the nanocomposite, turning a dried solid state into a turbid liquid state. The test tube was filled with ethanol (~5 mL) and shaken vigorously to remove excessive amine, PTCDA, and TOPO from the solution. The test tubes containing nanocomposite, ethanol, and chloroform were centrifuged at 14k rpm for 15 min. The hydrophobic nanocomposite was precipitated as pellets at the bottom of the test tubes. The supernatant was decanted and discarded. In most cases, three purification cycles were performed using this procedure. The nanocomposite precipitate was suspended into 2–3 mL of fresh chloroform for future use. Figure 2 shows the solution-phase UV-Vis and fluorescence of QDs and PTCDA. The confirmation of CdSe QDs was also confirmed using XRD measurements (Figure S1).

## 4. Summary

In this work, we have synthesized and characterized the QD–PTCDA–MWCNTs nanocomposite in one-pot synthesis for the fabrication of BHJSCs. The interaction between QDs and MWCNTs through a molecular linker (PTCDA) was studied and characterized using spectroscopic and microscopic techniques. Whereas the FTIR analysis confirmed the covalent bonds between QDs and PTCDA, π–π stacking were formed between PTCDA and MWCNTs. Thus, these interactions led to about two orders of magnitude enhancement in the QD concentration on the MWCNTs as compared to nanocomposites created without PTCDA. Spectroscopic and microscopic characterization techniques were used to establish the nanoscale heterojunction interface in the nanocomposites. The PCE of the devices fabricated with QD–PTCDA–MWCNTs nanocomposite exhibited three times that of devices fabricated in the absence of PTCDA. With further optimization of the material properties and processing parameters, there are opportunities for further improvements in the performance of the BHJSCs fabricated with QD–PTCDA–MWCNTs nanocomposites synthesized in a single pot.

## Figures and Tables

**Table 1:** IR table of QDs, PTCDA, and QD–PTCDA–MWCNT.

Material	Peak Position (cm^−1^)	Peak Assignment	References
QD	2917–2848	C–H stretching	[33]
	1590	N–H bending	
	1452	C–H bending	
PTCDA	3700–2800 (broad)	O–H and C–H stretching	[29]
	1770 (sharp)	C=O from cyclic anhydride	
	1400–1600 (sharp)	C=C stretching	
QD–PTCDA–MWCNT	2911–2848 (sharp)	C–H stretching	[34,35]
	1664 (sharp)	C=O amide stretching	
	1592 (sharp)	N–H bending	
	1460 (sharp)	C–H bending	

**Table 2:** *K_sv_* values for different donor–acceptor pairs.

Donor	Acceptor	*K_sv_* (L mg^−1^)
QD	MWCNT	0.16
QD–PTCDA	MWCNT	1.09
PTCDA	MWCNT	1.05
QD	PTCDA	0.25

## Data Availability

Data is available from the authors with a reasonable request.

## Supplementary Materials

The following supporting information can be downloaded at: https://www.mdpi.com/article/10.3390/molecules28237702/s1, Figure S1: XRD of the CdSe QDs.; Figure S2: TEM of the unfunctionalized QDs.
