# Supplementary material for: One-Pot Synthesis of Semiconducting Quantum Dots–Organic Linker–Carbon Nanotubes for Potential Applications in Bulk Heterojunction Solar Cells
Source: Molecules. 2023 Nov 22;28(23):7702. doi: 10.3390/molecules28237702 (PMC10707941; doi:10.3390/molecules28237702)
Supplement: Supplementary file 1 [file molecules-28-07702-s001.zip › molecules-2641520-supplementary.pdf]

Supplementary Information

One-Pot Synthesis of Semiconducting Quantum Dots–Organic Linker–Carbon Nanotubes for Potential Applications in Bulk Heterojunction Solar Cells

Mallika Dasari [1], Baleeswaraiah Muchharla [2], Saikat Talapatra [2,*] and Punit Kohli [1,*]

[1] School of Chemical and Biomolecular Sciences, Southern Illinois University, Carbondale, IL 62901, USA; mallikachem14@gmail.com
[2] School of Physics and Applied Physics, Southern Illinois University, Carbondale, IL 62901, USA; balu167@siu.edu
* Correspondence: saikat@siu.edu (S.T.); pkohli@chem.siu.edu or kohlip@siu.edu (P.K.)

Figure S1: XRD of the CdSe QDs.
Figure S2: TEM of the unfunctionalized QDs.

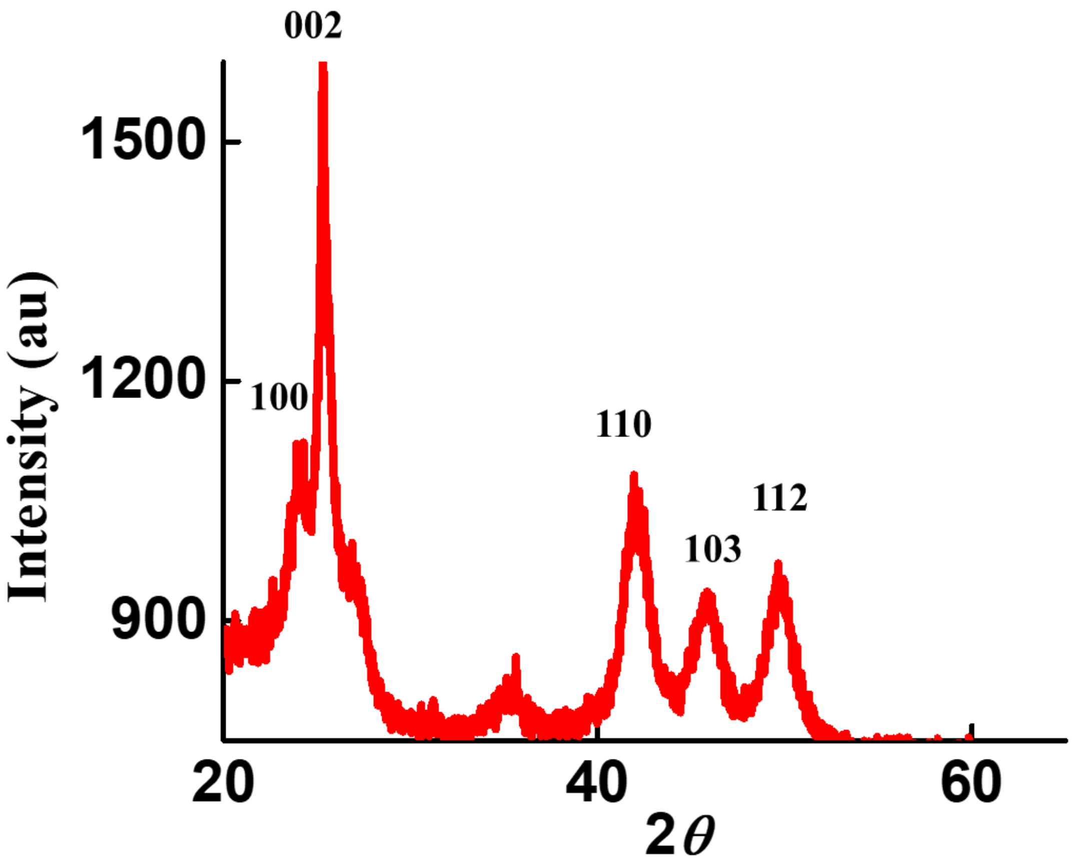

Figure 1S. XRD of the CdSe QDs.

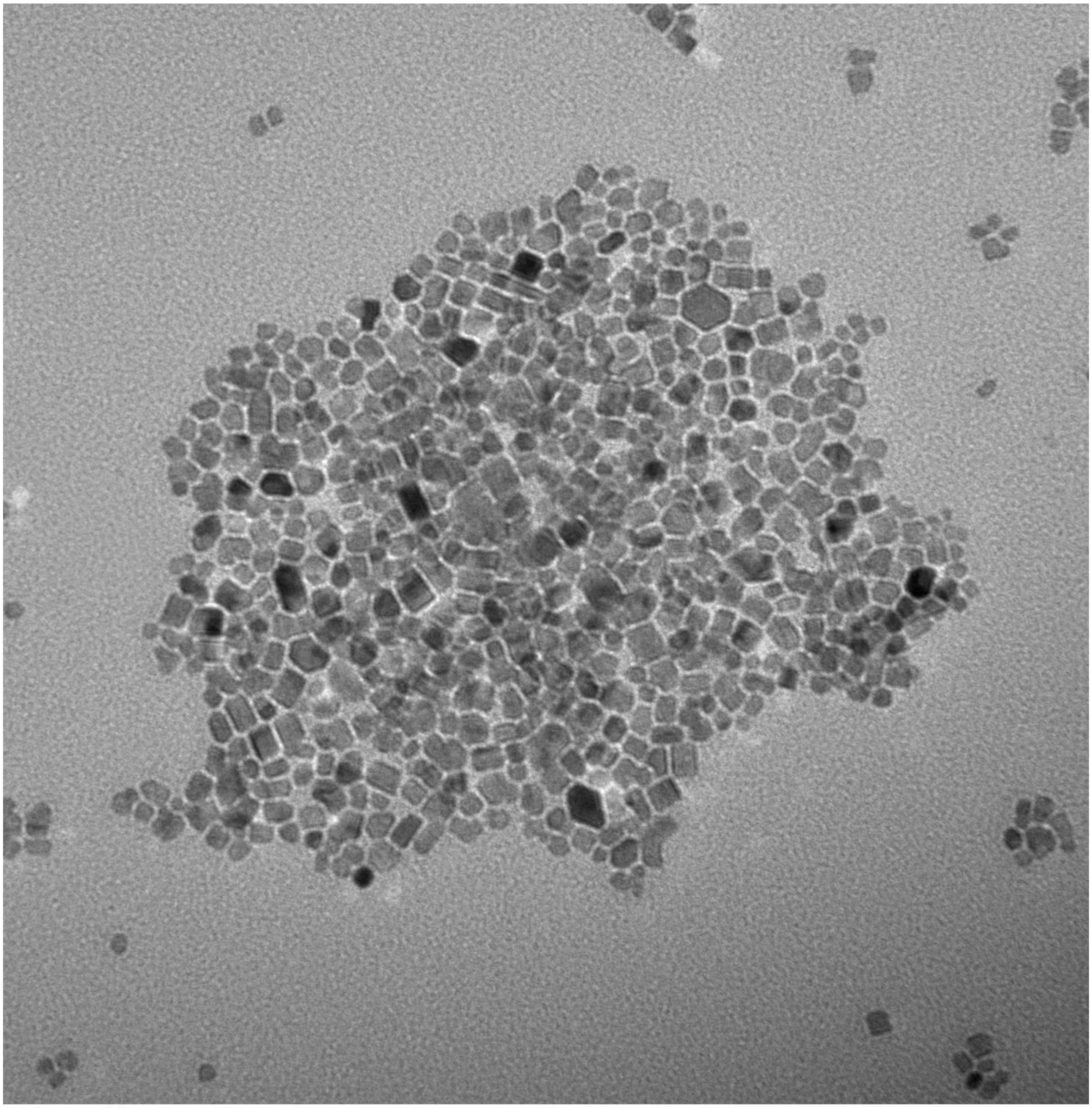

Figure 2S. TEM of the unfunctionalized CdSe QDs.
